# Comprehensive Small RNA-Seq of Adeno-Associated Virus (AAV)-Infected Human Cells Detects Patterns of Novel, Non-Coding AAV RNAs in the Absence of Cellular miRNA Regulation

**DOI:** 10.1371/journal.pone.0161454

**Published:** 2016-09-09

**Authors:** Catrin Stutika, Mario Mietzsch, Andreas Gogol-Döring, Stefan Weger, Madlen Sohn, Wei Chen, Regine Heilbronn

**Affiliations:** 1 Charité Medical School, Campus Benjamin Franklin, Institute of Virology, Berlin, Germany; 2 Technische Hochschule Mittelhessen, Gießen, Germany; 3 Max-Delbrück-Centrum für Molekulare Medizin, Berlin Institute for Medical Systems Biology, Laboratory for Functional Genomics and Systems Biology, Berlin, Germany; University of Kansas Medical Center, UNITED STATES

## Abstract

Most DNA viruses express small regulatory RNAs, which interfere with viral or cellular gene expression. For adeno-associated virus (AAV), a small ssDNA virus with a complex biphasic life cycle miRNAs or other small regulatory RNAs have not yet been described. This is the first comprehensive Illumina-based RNA-Seq analysis of small RNAs expressed by AAV alone or upon co-infection with helper adenovirus or HSV. Several hotspots of AAV-specific small RNAs were detected mostly close to or within the AAV-ITR and apparently transcribed from the newly identified anti-p5 promoter. An additional small RNA hotspot was located downstream of the p40 promoter, from where transcription of non-coding RNAs associated with the inhibition of adenovirus replication were recently described. Parallel detection of known Ad and HSV miRNAs indirectly validated the newly identified small AAV RNA species. The predominant small RNAs were analyzed on Northern blots and by human argonaute protein-mediated co-immunoprecipitation. None of the small AAV RNAs showed characteristics of bona fide miRNAs, but characteristics of alternative RNA processing indicative of differentially regulated AAV promoter-associated small RNAs. Furthermore, the AAV-induced regulation of cellular miRNA levels was analyzed at different time points post infection. In contrast to other virus groups AAV infection had virtually no effect on the expression of cellular miRNA, which underscores the long-established concept that wild-type AAV infection is apathogenic.

## Introduction

Adeno-associated viruses (AAV) belong to the family of parvoviruses and possess a single-stranded DNA genome of approximately 4.7 kb. A characteristic feature of AAV is its biphasic life cycle. In the absence of a helper virus AAV establishes latent infection and integrates into the host genome or persist as nuclear episome [1–3]. Co-infection with a helper virus, e.g. adenovirus or herpesvirus results in AAV replication and progeny formation [4–8]. AAV type 2 represents the best-studied serotype and is commonly accepted as AAV prototype. The AAV2 genome contains two major open reading frames, *rep* and *cap*, which are flanked by hairpin-structured, 145 bp inverted terminal repeats (ITRs) at either end [9]. The *rep* gene encodes the four regulatory proteins Rep78 and Rep68, and N-terminally truncated versions thereof, called Rep52 and Rep40, respectively. The AAV capsid proteins VP1, VP2 and VP3 are encoded by the *cap* gene. Furthermore, *cap* encodes the assembly activating protein (AAP) by use of an alternative open reading frame [10]. Early AAV2 transcription mapping only defined transcripts derived from the coding AAV positive (+) strand. These mRNAs initiate at the p5, p19 or p40 promoters, named according to their relative positions on the AAV2 genome. In a total RNA-Seq analysis, we have recently discovered transcription on the AAV negative (-) strand opposite to the p5 promoter, indicative of non-coding RNA species [11]. In addition, we have identified p40 promoter-associated short non-coding transcripts on the (+) strand relevant for the inhibition of adenovirus replication [12]. Apparently, non-coding RNA species are involved in the regulation of the AAV life cycle.

Small non-coding RNAs represent a growing class of diverse, regulatory RNAs. Of these, microRNAs (miRNAs) and short interfering RNAs (siRNAs) represent the best-characterized species. These RNAs are approximately 22 nucleotides in length and are processed by the cellular enzyme Dicer from longer double-stranded RNA precursors that form a distinctive, secondary RNA structure [13, 14]. One strand of the processed RNA duplex is loaded into the RNA-induced silencing complex (RISC) allowing recognition of the mRNA target sequence. Mammalian miRNAs and siRNAs typically represent posttranscriptional inhibitors by specifically binding to a target RNA leading to translational repression or mRNA degradation [15, 16]. Less well characterized classes of small regulatory RNAs have since been described, whose functions are largely unknown. Of these, tRNA-derived fragments (tRFs) or microRNA-offset RNAs (moRs) have been suggested to play a miRNA-like role in posttranscriptional gene silencing [17, 18]. Others, such as promoter-associated RNAs (paRNAs) appear to be specifically involved in regulating promoter activity [19].

Most DNA virus genera and also certain RNA viruses express small non-coding RNAs [20, 21], but often the molecular function has not been fully defined. Adenovirus (Ad) generates miRNAs processed from the longer structured virus-associated RNAs, VA-RNA I and II. The VA-RNAs themselves are described to suppress the cellular RNA interference (RNAi) pathway by interfering with the activity of Dicer [22]. For the VA-RNA derived miRNAs (mivaRNAs) cellular target genes were identified, some of which are involved in the regulation of cellular gene expression [23, 24]. The exact role of the Ad mivaRNAs for the adenovirus life cycle has not yet been defined. Most members of the herpesvirus family encode clusters of miRNAs, which are differentially expressed during latent or lytic infection. During HSV1 latency only a single abundant viral transcript is expressed. This latency-associated transcript (*LAT*) represents a non-coding RNA that serves as a precursor for several miRNAs [25], assumed regulators of HSV1 latency. Of the 18 well described HSV1 miRNAs, a role during the herpesviral life cycle has only been determined for three: miR-H2 and miR-H6 repress the major HSV1 transcriptional activators ICP0 and ICP4, respectively, and thus maintain the latent state [25], and miR-H4 has been shown to target the HSV1 pathogenicity factor ICP34.5 [26]. For certain herpesviruses microRNA-offset RNAs have been identified, whose functions are presently unknown [27, 28].

AAV represents one of the very few DNA viruses for which small regulatory RNAs have not yet been described. Its bipartite life cycle combined with the hairpin-structured AAV-ITRs reminiscent of the structure of miRNA precursors led to the hypothesis that small RNA species with regulatory function might be expressed by AAV as well. In the first comprehensive Illumina-based small RNA-Seq analysis of AAV-infected cells we discovered and validated several classes of previously unknown small non-coding RNAs.

## Materials and Methods

### Cell culture and viruses

HEK 293 and HeLa cells were obtained over 30 years ago from ATCC and passaged since as described [29]. Human adenovirus type 2 (Ad2) was propagated and quantified in 293 cells. The HSV1 KOS strain was produced in Vero cells and titrated by plaque assay.

### AAV2 production, purification and quantification

For AAV2 production HEK 293 cells were seeded at 30% confluency and transfected 24 hours later with pTAV2-0 [30] and pHelper as described [2]. AAV2 was purified from benzonase-treated, cleared freeze-thaw supernatants by one-step AVB sepharose affinity chromatography and quantified by Light-Cycler PCR as described [31] with primers specific for AAV2 *rep* (Rep2-Fwd: AGAAGGAATGGGAGTTGCCG and Rep2-Rev: TCTGACTCAGGAAACGTCCC). AAV2 infectious titers were determined by end point dilutions on Ad2 infected HeLa cells [32].

### Virus infection

HeLa cells were seeded at a density of 30% and infected 20 hours later with AAV2 wild-type (MOI 250), Ad2 (MOI 25) or HSV1 (MOI 10), or indicated combinations thereof.

### RNA extraction

Total RNA was extracted 8 hpi and 27 hpi from AAV2 infected cells in presence or absence of HSV1 or Ad2, respectively. Total RNA was isolated using TRIzol Reagent (Ambion) according to the manufacturer’s protocol. Subsequently, RNA samples were treated with RNase-free Turbo DNase (Ambion), followed by phenol-chloroform extraction and precipitation. RNA quality and integrity was verified on 0.8% agarose gels and on bioanalyzer (Agilent). Total RNA samples of proven high quality were used for small RNA library generation and Northern blot analysis.

### Northern blot analysis

For electrophoresis 15% polyacrylamide gels containing 8 M urea and 0.5x MOPS running buffer (10x MOPS is 200 mM MOPS, 50 mM sodium acetate and 10 mM EDTA, pH 7.0) were used. Prior to loading of the samples the gel was pre-run at 100 V for 30 min. RNA samples were treated using the FDF-PAGE method as described [33]. In short, equal amounts of total RNA (5 μg to 25 μg per lane) in a volume of 4 μl were mixed with 11 μl FDF buffer (2.75 μl formaldehyde (37%), 7.5 μl formamide (>99.5%) and 0.75 μl 10x MOPS buffer), incubated at 55°C for 15 min and subsequently mixed with 2 μl of 10x dyes (0.05% [w/v] xylene cyanol and 0.05% [w/v] bromphenol blue in nuclease-free water) before loading. In addition 1 μl of microRNA ladder (NEB) and 5 μl of low range ssRNA ladder (NEB) were loaded onto the gel. Electrophoresis was run at 150–200 V until the bromphenol blue dye reached about 2 cm at the bottom of the gel. After electrophoresis, gels were stained in ethidium bromide (10 μg/ml) to visualize the RNA size markers. RNAs were transferred to a positively charged nylon membrane (GeneScreen Plus, PerkinElmer) at 1 mA/cm^2^ for 1.5 h or overnight by electroblotting, and cross-linked by UV (Stratagene) or EDC for 2 h at 60°C as described [34]. The membranes were pre-hybridized for 2 h in pre-warmed hybridization buffer (5x SSC [10x SSC is 1.5 M NaCl plus 0.15 M sodium citrate], 1% SDS and 1x Denhardt´s Solution [100x Denhardt´s Solution is 2% [w/v] BSA, 2% [w/v] Ficoll 400 and 2% [w/v] Polyvinylpyrrolidone]). DNA-oligonucleotides (MWG Eurofins) or DNA/LNA-oligonucleotides (Exiqon) [35] with sequences complementary to sRNAs (see S1 Table) were 5’ end labeled with [γ-^32^P]-ATP (Hartmann Analytic), and purified on a Bio-Gel P-10 column (Bio-Rad). Membranes were hybridized for 16 hours at 37°C or at 55–65°C with the labeled DNA- or DNA/LNA-probes, respectively. Subsequently, membranes were washed three times for 10 min with low stringency buffer (2x SSC / 0.1% SDS), followed by two washing steps at higher stringency (1x SSC / 0.1% SDS) for 5 min at 37°C or at 55–65°C for membranes hybridized with DNA- or DNA/LNA-probes, respectively. Membranes were exposed to X-ray films between intensifying screens at -80°C overnight or longer.

### Co-immunoprecipitation (Co-IP) of human argonaute protein complexes

Co-immunoprecipitation was carried out as described [23]. In brief, uninfected or infected HeLa cells were washed twice with ice-cold PBS 27 hpi and lysed in cell lysis buffer containing 25 mM Tris-HCl (pH 8.0), 150 mM NaCl, 2 mM MgCl_2_, 0.5% Nonidet P40, 5 mM dithiothreitol (DTT), protease inhibitor (cOmplete Tablets, Mini Easypack, Roche) and 40 U/ml RNase inhibitor (Recombinant RNasin Ribonuclease Inhibitor, Promega) for 30 min at 4°C on an overhead tumbler. Cell debris was removed by centrifugation at 20,000 g for 30 min at 4°C. For co-immunoprecipitation, 100 μl Protein G-Agarose beads (Roche) per preparation were washed three times in ice-cold nuclease-free PBS and subsequently resuspended in 1 ml nuclease-free PBS supplemented with protease inhibitor cocktail. 15 μg of anti-pan Ago mAb clone 2A8 (Merck Millipore) or anti-Rep mAb 76–3 (Progen) were added to the pre-washed beads, respectively, and incubated for 6 hours at 4°C under constant rotation. Antibody-coupled beads were blocked at 4°C over night in blocking solution consisting of nuclease-free PBS containing 1.2 mg/ml BSA, 0.6 mg/ml yeast tRNA (Roche) and protease inhibitor cocktail. The beads were washed three times in ice-cold blocking solution and once with cell lysis buffer. For immunoprecipitation, the cell lysates were incubated with the prepared antibody-coupled beads under constant rotation at 4°C over night. Following, the beads were washed twice with cell lysis buffer supplemented with BSA and tRNA, three times with high salt washing buffer (25 mM Tris-HCl (pH 8.0), 900 mM NaCl, 2 mM MgCl_2_, 1% Nonidet P40, 5 mM DTT, protease inhibitor cocktail, 40 U/ml RNase inhibitor, BSA and tRNA) and twice with low salt washing buffer (25 mM Tris-HCl (pH 8.0), 150 mM NaCl, 2 mM MgCl_2_, 0.05% Nonidet P40, 5 mM DTT, protease inhibitor cocktail, 40 U/ml RNase inhibitor, BSA and tRNA). Co-immunoprecipitated RNAs were isolated from the beads using the TRIzol Reagent (Ambion) followed by RNA precipitation and purification.

### Small RNA library preparation and Illumina Sequencing

1 μg of each of the eight different total RNA samples (cells, AAV2, AAV2 + HSV1, HSV1 extracted 8 hpi and cells, AAV2, AAV2 + Ad2, Ad2 extracted 27 hpi) were subjected to small RNA library generation using the TruSeq Small RNA Sample Prep Kit according to the manufacturer’s protocol (Illumina). Briefly, after ligation of the RNA 3’- and the RNA 5’ adapter, total RNA samples were reverse transcribed to cDNA libraries and subsequently PCR-amplified. The PCR products were run on 6% PAGE gels and bands of adapter-ligated DNA libraries between 145 and 160 basepairs (bp) were excised from the gel and purified. These bands correspond to RNAs of a length of approximately 20 to 35 nucleotides. Subsequently, the libraries were validated by bioanalyzer, pooled and sequenced on a HiSeq 2000 platform (multiplexed 1x51+7).

### Processing of sequencing reads

The sequencing reads were demultiplexed and the sequencing adapters were removed. Reads shorter than 16 bp were discarded; the remaining reads were mapped without mismatch to the human genome (hg19), transcriptome (generated from RefSeq annotations) and pre-miRNA sequences (downloaded from the miRBase [36], http://www.mirbase.org) using Bowtie [37]. The unmapped reads were further aligned to HSV1 (accession number NC_001806.1), Ad2 (accession number AC_000007.1) and AAV2 (accession number NC_001401.2) reference genomes. Additional sequence mapping was performed gradually allowing zero, one and two mismatches (see S2 Table).

### Database of microRNAs, secondary structure prediction and miRNA target prediction

Known human, HSV1 and Ad microRNAs were obtained from miRBase, the database of microRNAs (http://www.mirbase.org/). For secondary structure prediction of possible precursor RNAs the Vienna RNAfold WebServer was used (http://rna.tbi.univie.ac.at/cgi-bin/RNAfold.cgi). To search for putative target sites of described human miRNAs the miRNA target prediction tool “Target Scan” was applied (http://www.targetscan.org).

## Results

### Small RNA-Seq library preparation

To identify AAV2-derived small RNA species we performed Illumina small RNA next generation sequencing. HeLa cells were infected with AAV2 alone or co-infected with AAV2 and the helper viruses Ad2 or HSV1, respectively. Uninfected, Ad2 and HSV1 infected cells served as controls. Total RNAs were extracted at 8 hpi after HSV1 infection or 27 hpi after Ad2 infection. Together, two sets of four total RNA samples were generated, each representing the latent and lytic AAV2 infection state including controls. All samples contained highly intact RNA as validated by bioanalyzer displaying RNA integrity numbers (RIN) higher than 8.90. Using the Illumina TruSeq small RNA sample preparation kit, libraries of RNAs with sizes between 20 to 35 nucleotides were generated. The integrity of the libraries was re-checked immediately prior to sequencing on the Illumina HiSeq 2000 platform.

### Illumina small RNA-Seq next generation sequencing

For each data set between 18.0 and 24.9 million RNA reads were obtained by Illumina small RNA-Seq analysis (Table 1). The majority of these reads (>88%) corresponded to RNAs with a size of at least 16 nucleotides (nt). Based on these, a total of 50 to 85% could be assigned to sequences of the human genome without allowing any mismatch. Due to the inherent error rate of Illumina sequencing, higher numbers of reads could be assigned to the human and viral genomes when allowing one and two mismatches (S2 Table). While the numbers per mapped read increased, this did not lead to the detection of further small RNAs. In all of the 8 data sets a significant portion of reads could be mapped to known human miRNAs (Table 1). In the absence of a helper virus very low numbers of small AAV2-specific RNA reads were detectable in AAV2 infected cells. Their numbers were increased by more than 200-fold in presence of HSV1 or Ad2 accounting for 0.7 to 1.4% of total small RNA reads displaying at least 16 nt in size (Table 1). The consistently lower read numbers in the HSV data set may be due to the short HSV replication cycle and consequently earlier time point of cell harvest (8 hpi versus 27 hpi). Similar to AAV2, HSV1 showed a low number of specific small RNAs (0.5 to 0.6% of total reads), whereas the number of small RNAs mappable to Ad2 was significantly higher (14.1 to 22.0% of total reads). On the other hand, Ad2-specific read numbers were significantly reduced in presence of AAV2, presumably reflecting the known inhibitory effect of AAV on adenovirus replication [38, 39]. Furthermore, in the presence of Ad2 human miRNAs were slightly less abundant compared to those in the other data sets (Table 1) confirming the recent finding that certain human miRNAs are dramatically deregulated during adenovirus infection [40]. In addition, adenovirus was shown to block the RNAi processing machinery in order to generate its own miRNAs late during infection [22]. In the following all further analyses were performed using zero mismatches per mapped read (Table 1).

**Table 1:** Small RNA-Seq—Assignment of the reads to the species from which they originate.

Data set	Total no. of reads	No. (%) of reads ≥ 16 nt	No. (%) of human reads^a^	No. (%) of small reads assigned to:				No. (%) of unknown reads^b^
				Human miRNAs	AAV2	Ad2	HSV1	
**Cells (27 hpi)**	19,395,459	17,429,392 (89.9)	14,653,485 (84.1)	4,793,871 (27.5)	17 (<0.1)	348 (<0.1)	6 (<0.1)	809,469 (4.6)
**AAV2 (27 hpi)**	18,085,432	16,228,519 (89.7)	13,331,538 (82.1)	5,840,779 (36.0)	1,193 (<0.1)	1,209 (<0.1)	9 (<0.1)	1,037,657 (6.4)
**AAV2 + Ad2 (27 hpi)**	21,333,021	18,814,505 (88.2)	11,238,151 (59.7)	3,307,157 (17.6)	270,577 (1.4)	2,644,519 (14.1)	12 (<0.1)	2,142,730 (11.4)
**Ad2 (27 hpi)**	24,841,074	24,202,341 (97.4)	12,151,399 (50.2)	5,129,522 (21.2)	24 (<0.1)	5,332,713 (22.0)	14 (<0.1)	6,079,458 (25.1)
**Cells (8 hpi)**	21,851,591	20,985,998 (96.0)	17,475,849 (83.3)	6,899,774 (32.9)	15 (<0.1)	286 (<0.1)	13 (<0.1)	2,644,242 (12.6)
**AAV2 (8 hpi)**	20,375,107	20,022,269 (98.3)	16,464,292 (82.2)	7,551,710 (37.7)	160 (<0.1)	449 (<0.1)	10 (<0.1)	3,204,520 (16.0)
**AAV2 + HSV1 (8 hpi)**	23,031,470	20,904,023 (90.8)	16,445,457 (78.7)	7,202,911 (34.5)	146,807 (0.7)	3,933 (<0.1)	108,860 (0.5)	2,071,519 (9.9)
**HSV1 (8 hpi)**	22,391,766	20,895,686 (93.3)	16,671,702 (79.8)	9,745,884 (46.6)	14 (<0.1)	326 (<0.1)	124,055 (0.6)	2,603,509 (12.5)

^a^Included are reads mapped to the human genome, transcriptome and miRNAs.

^b^Included are reads ≥ 16 nt that were unmappable during sequence mapping allowing zero mismatches.

### Small RNA read mapping to the AAV2 (+) and (-) strand

**Fig 1:**
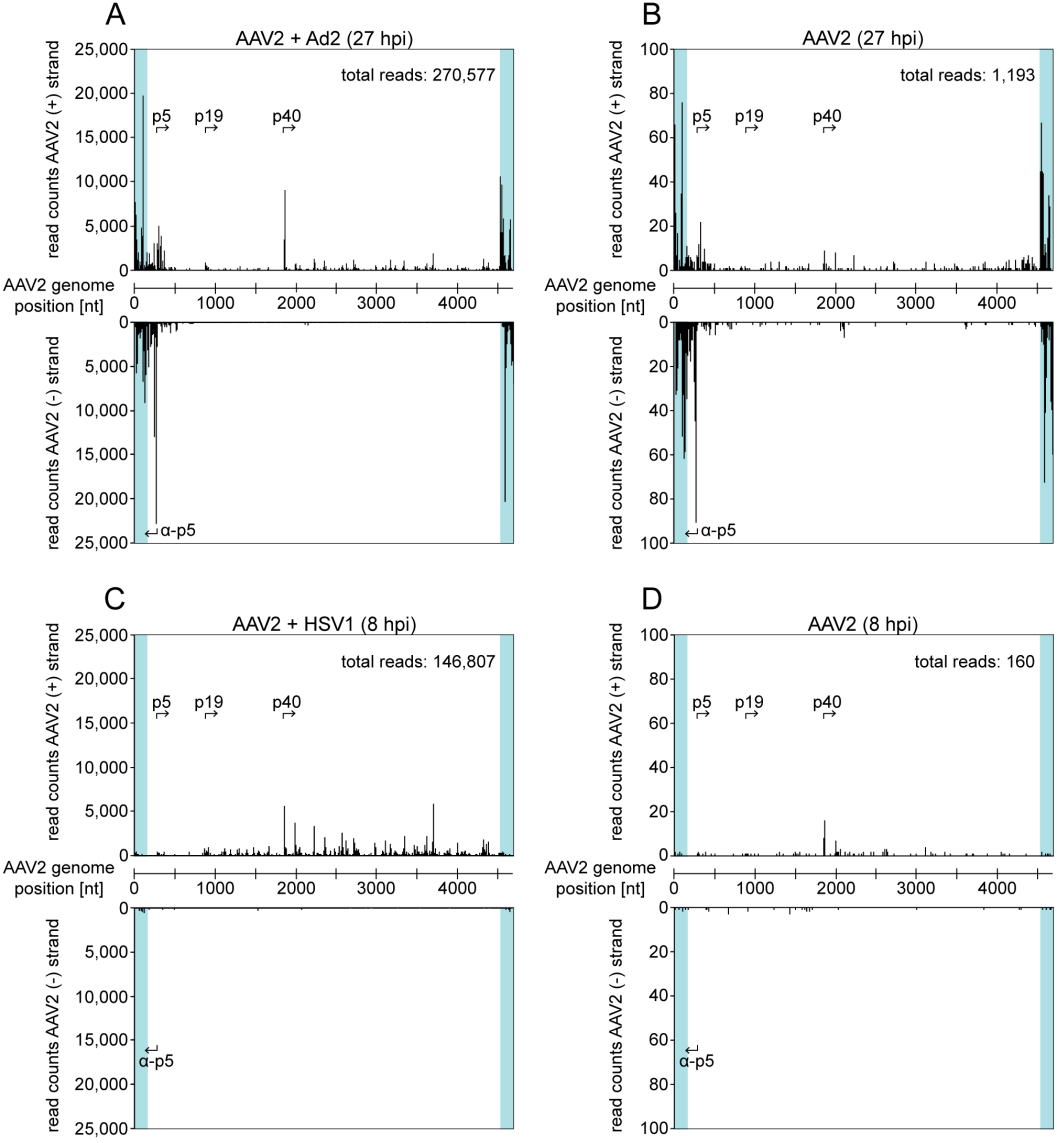
Small RNA-Seq: Read mapping of small AAV2-specific RNAs to either strand of the genome. (A) Mapping of AAV2-specific reads identified by the small RNA-Seq analysis in AAV2/Ad2 co-infected HeLa cells 27 hours p.i. (hpi). The AAV2 genome scale is displayed in the center. Reads mapped to the AAV2 (+) strand are displayed above and those mapped to the AAV2 (-) strand below the genome, read counts are shown in intervals of 10 nucleotides. The ITR regions are highlighted in turquoise. The positions of the known AAV promoters on either strand are indicated by arrows. The total read count is indicated. Note the ambiguous assignment of reads mapping to the ITRs. (B) Mapping of small AAV2-specific reads in AAV2 infected HeLa cells 27 hpi, displayed as in (A). (C) Mapping of small AAV2-specific reads in AAV2/HSV1 co-infected HeLa cells 8 hpi, displayed as in (A). (D) Mapping of small AAV2-specific reads in AAV2 infected HeLa cells 8 hpi, displayed as in (A). Note the different scales of read counts in (A to B) and (C to D).

The population of AAV2-specific small RNA reads for each data set was mapped to the wild-type AAV2 genome. For a better overview RNA reads were initially counted in closed intervals of 10 nucleotides. In the presence of Ad2, AAV2 infected cells showed a high number of small RNAs located within the ITRs (Fig 1A). Since small RNAs located in the ITRs cannot be mapped unambiguously to one ITR, they were assigned to either end. A significant amount of reads mapped to the region opposite of the p5 promoter (anti-p5) on the AAV (-) strand and to a region close to the p40 promoter on the AAV (+) strand (Fig 1A). The transcription profile of small AAV2 RNAs (27 hpi) was comparable in cells with and without Ad2 infection, but the absolute read numbers differ by a factor of around 200. However, no accumulation of p40 promoter-associated reads could be observed in the absence of Ad2 (Fig 1B). In the presence of HSV1 the AAV transcription profile of small RNAs was different to that observed in the presence of Ad2 (Fig 1C). Neither the ITR nor the AAV (-) strand showed an accumulation of small RNA reads, but similarly to Ad2 co-infected cells, there was indication of a RNA hotspot close to the p40 promoter (compare Fig 1A and 1C). Additional small RNA reads were located downstream of the p40 promoter (Fig 1C). RNA harvested from AAV infected cells (8 hpi) showed hardly any AAV2-specific reads (Fig 1D). Obviously, AAV transcription in the absence of a helpervirus starts delayed (compare Fig 1A to 1B and 1C to 1D).

### Mapping of small RNA reads to the Ad2 and HSV1 genomes

**Fig 2:**
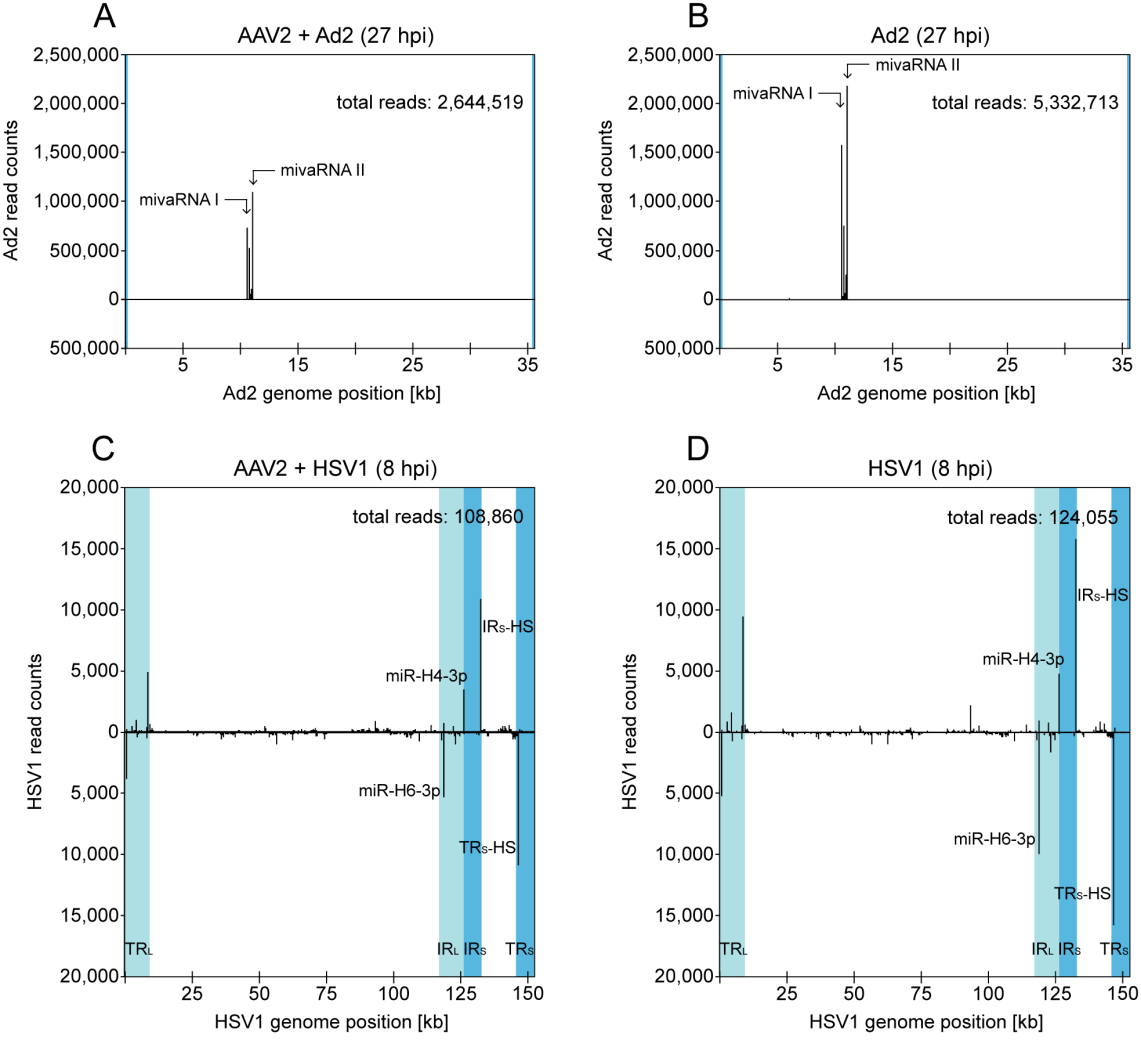
Small RNA-Seq: Mapping of small RNA reads to the genomes of Ad2, or HSV1. (A) Mapping of Ad2 reads identified by the small RNA-Seq analysis to the Ad2 genome in AAV2/Ad2 co-infected HeLa cells 27 hpi. The Ad2 genome scale is displayed in the center. Reads mapped to the Ad2 (+) strand are displayed above and to the Ad2 (-) strand below, read counts are shown in intervals of 10 nucleotides. Terminal repeats flanking the adenoviral genome are highlighted in turquoise. Reads assigned to the described adenoviral mivaRNAs I and II are indicated by arrows. The total read count of small RNAs is indicated. (B) Mapping of small Ad2 reads in Ad2 infected HeLa cells 27 hpi, displayed as in (A). (C) Mapping of HSV1 reads identified by the small RNA-Seq analysis to the HSV1 genome (KOS strain) in AAV2/HSV1 co-infected HeLa cells 8 hpi, displayed as in (A), but in intervals of 100 nucleotides. Terminal (TR) and internal repeat (IR) regions within the HSV1 genome are highlighted in shades of turquoise. Small RNAs of high read frequencies were assigned to known HSV1 miRNAs according to miRBase. A small RNA with very high read counts was designated as IR_S_-HS / TR_S_-HS due to its location within these repeat regions. Note the ambiguous assignment of reads mapping to the repeat regions. (D) Mapping of small HSV1 reads in HSV1 infected HeLa cells 8 hpi, displayed as in (C). Note the different scales of read counts in Ad2 infected cells (A, B) and HSV1 infected cells (C, D).

Comparative analysis of Ad2 and HSV1 as helper viruses for productive AAV2 replication allowed the validation of small RNA-Seq data by analysis of known Ad or HSV encoded miRNAs, respectively. Indeed, the majority of Ad2-specific small RNA reads (>95%) could be assigned to the adenoviral mivaRNAs in the absence, as well as in the presence of AAV2 (Fig 2A and 2B). These small RNAs, processed from the longer structured VA RNA I and II, are expressed in lytic, as well as in persistently infected cells [41, 42]. Co-infection with AAV2 reduced the numbers of mivaRNA I and II by half, likely explained by the repressive activity of co-replicating AAV2. Similarly, many of the HSV1-specific small RNA reads could be assigned to known HSV1 encoded miRNAs in the data sets of HSV1 infected cells in the presence or absence of AAV2 (Fig 2C and 2D). Since small RNA reads within repeat regions cannot be unambiguously mapped, they were assigned to either HSV repeat as applied here for the small AAV RNAs. Prominent hotspots could be attributed to the miRNAs miR-H4-3p and miR-H6-3p located within the HSV1 repeat region IR_L_ (TR_L_) (Fig 2C and 2D). Furthermore, HSV1-miR-1 and -2 were moderately expressed with 300 and 700 reads, respectively, whereas HSV1-miR-3, -5, -11, -12 and -16 were hardly detectable (<100 reads). HSV miRNAs are differently expressed during latent and lytic infection in highly susceptible cell types [27]. In HeLa cells used here, a prominent small RNA hotspot was detected at HSV1 genome position 132,142 [+] or 146,091 [–] (Fig 2C and 2D, named IR_S_-HS / TR_S_-HS). This hotspot has also been reported in another study during early lytic HSV1 infection just recently [43].

### Detailed mapping of AAV2-specific small RNAs

**Fig 3:**
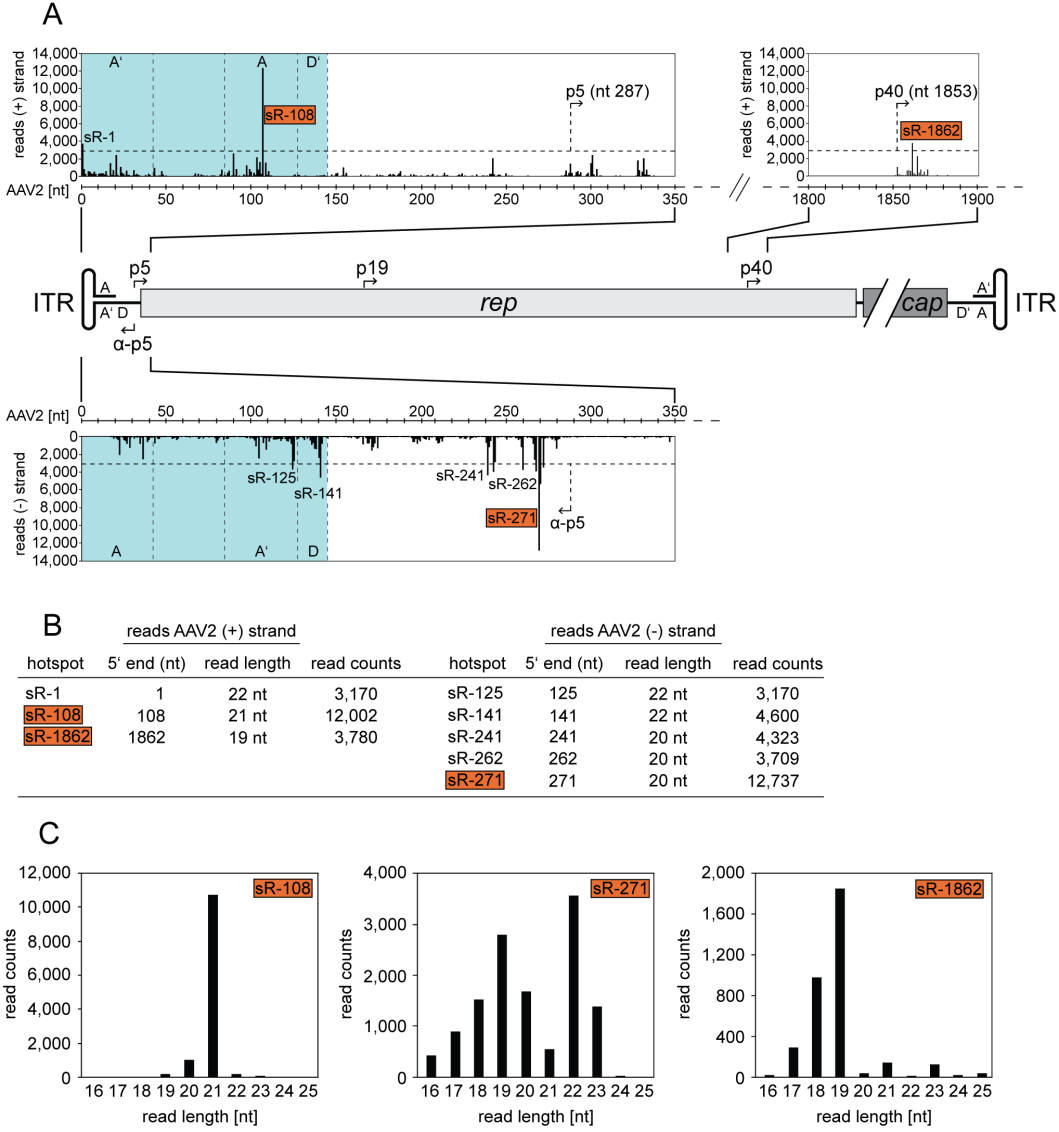
Small RNA-Seq: Detailed mapping of small AAV2-specific reads to both strands of the genome. (A) Detailed mapping of small AAV2-specific reads in regions of high read frequencies in AAV2/Ad2 co-infected cells from Fig 1A. A schematic representation of the AAV2 genome is displayed in the center. Read assignments to the (+) strand are presented above and to the (-) strand below the genome, with read counts displayed at single nucleotide level. A horizontal dashed line marks the threshold value set to 3,000 reads. Any small RNA (sR) above 3,000 read counts was defined as hotspot candidate, designated according to the 5’ starting nucleotide. Different genomic regions of the AAV2-ITRs (highlighted in turquoise) are separated by vertical dashed lines. Complementary regions of the hairpin-structured ITR are indicated by letters, e.g. A, A’; D, D’. Promoters on the AAV2 (+) or (-) strand are indicated by arrows. (B) Summary of small RNA hotspot candidates on the AAV2 genome, displayed in (A), designated according to their starting nucleotide (5’ end). Average read lengths are shown for the small RNAs. (C) Depicted are the detailed small RNA read length distributions for sR-108, sR-271 and sR-1862. Small RNA hotspots highlighted in red were validated further.

For more precise mapping of the small AAV2-specific RNAs the regions with high read counts were enlarged to single nucleotide level in the data set of AAV2/Ad2 co-infected cells (Fig 3A). The 5’ end of any small RNA was defined as starting nucleotide used henceforth as nomenclature for these RNAs. Even on a single nucleotide level hotspots of small RNAs were detected. The threshold was arbitrarily set to 3,000 reads, which is approximately 100-fold above background. Only small RNAs above this threshold were defined as hotspots and analyzed further. Four hotspots were located within the AAV-ITRs, sR-1 and sR-108 on the AAV (+) strand, and sR-125 and sR-141 on the AAV (-) strand (Fig 3A). Due to the ambiguous character of the ITRs sR-1 on the AAV (+) strand is equivalent to sR-125 on the AAV (-) strand with 3,170 reads (Fig 3B). Furthermore, sR-1, sR-108 and sR-141 can be mapped to either ITR on the AAV (+) or (-) strand (see S3 Table). In fact, sR-108 represented one of the two most abundant small RNAs with 12,002 read counts (Fig 3B) with a predominant mean read length of 21 nt (Fig 3C). The other prominent hotspot, displaying sR-271, was located downstream of the newly identified anti-p5 promoter on the AAV (-) strand [11] with 12,737 reads and showed varying read lengths between 16 and 23 nt (Fig 3C). Furthermore, additional minor hotspots clustered in that region (Fig 3A and 3B). Another hotspot, displaying sR-1862, was located just downstream the transcription start site (TSS) of the p40 promoter with 3,780 read counts. This particular small RNA showed a read length between 18 and 19 nt (Fig 3C). All hotspots displayed an average read length between 19 and 22 nt, typically seen for small non-coding regulatory RNAs (Fig 3B). The overall expression of the AAV2-specific small RNAs was comparable to that of HSV1 small RNAs but lower than that of Ad mivaRNAs. For AAV2/HSV1 infected cells the small AAV2-specific reads were of low abundance and mostly below the threshold of 3,000 reads. A list of the top hundred AAV-specific small RNAs in the presence of the helper viruses Ad and HSV, respectively, is given in S3 Table, which includes data of genomic location, read counts, median read length and sequence of the respective small AAV RNAs.

### Validation of the AAV2-specific small RNA hotspots

**Fig 4:**
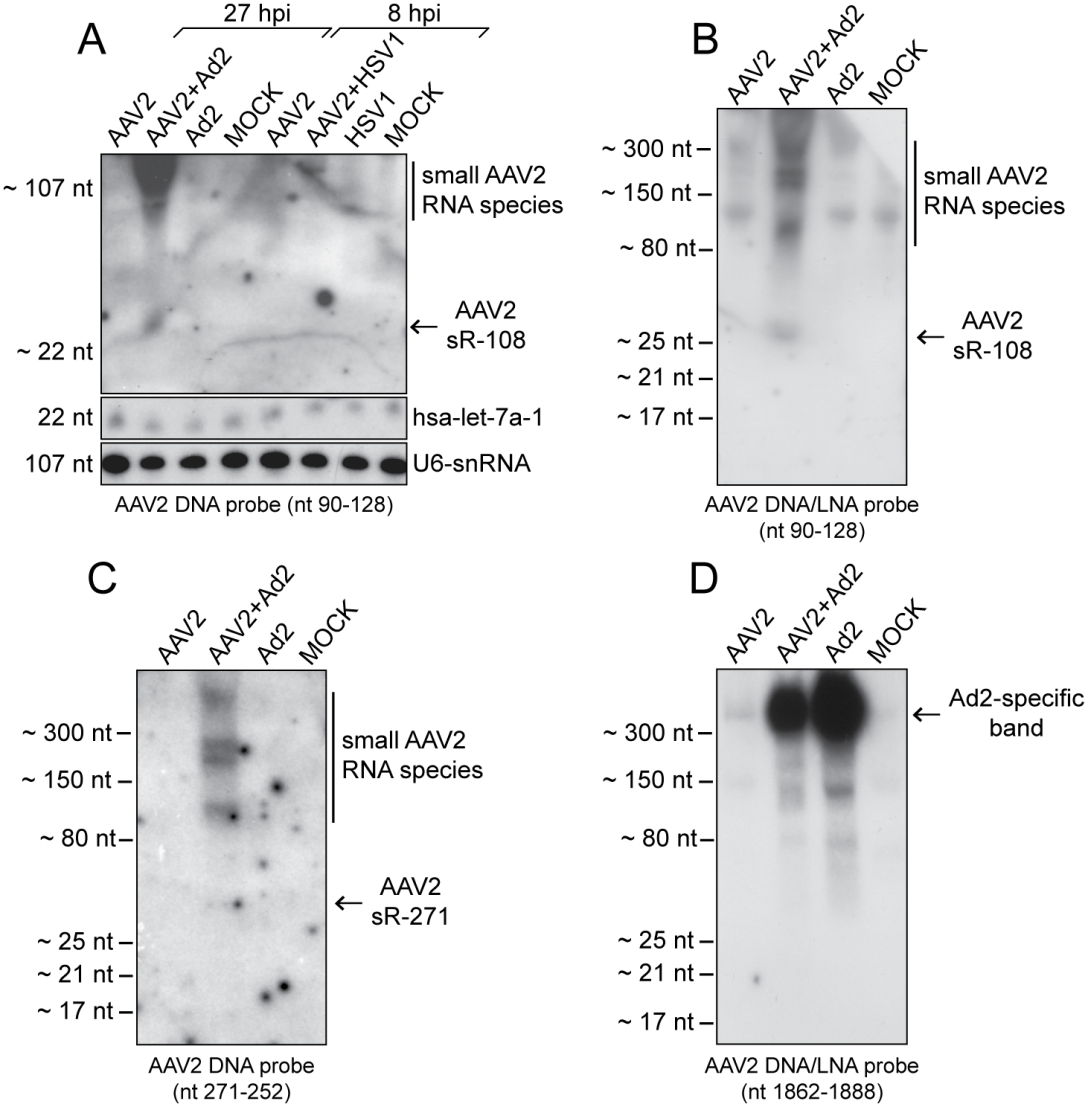
Detection of AAV2-specific small RNAs by Northern blot analysis. (A) Northern blot analysis of DNase treated total RNA extracts from HeLa cells, either none infected (Mock), or infected with AAV2, AAV2 and Ad2, AAV2 and HSV1, Ad2, or HSV1, harvested at the indicated time points. Total RNA extracts separated on 15% PAA-Urea gels, transferred to a positively charged membrane were hybridized with radiolabeled DNA probes directed against the depicted AAV2 small RNA sR-108, the human miRNA hsa-let-7a, or the cellular U6-snRNA, respectively. To the right detected small RNAs are indicated. (B) Experimental procedure as in (A) but RNAs were detected by a radiolabeled DNA/LNA probe directed against the AAV2 small RNA sR-108. (C) Experimental procedure as in (A) but RNAs were detected by a radiolabeled DNA probe directed against the AAV2 small RNA sR-271. (D) Experimental procedure as in (A) but RNAs were detected by a radiolabeled DNA/LNA probe targeting the AAV2 small RNA sR-1862. To the left, the molecular sizes of the bands of the miRNA ladder and the ssRNA ladder, respectively, are depicted.

To confirm the presence of AAV2-specific small RNAs by an alternative method, total RNA was harvested from AAV2 infected and AAV2/Ad2 co-infected HeLa cells and respective controls. RNA samples treated with RNase-free DNase were analyzed on Northern blots with specific probes directed against the most prominent AAV2-specific small RNAs. As internal controls, the human miRNA hsa-let-7a-1 and the splicosomal U6-snRNA were detected in all RNA extracts (Fig 4A) showing the high quality of the RNA samples. The highly abundant sR-108 located in the ITR of the AAV (+) strand was detected with a specific DNA probe (Fig 4A) and also with a specific DNA/LNA probe (Fig 4B) only in extracts harvested from AAV2/Ad2 co-infected cells, but not in AAV2/HSV1 co-infected cells (Fig 4A), which is in agreement with the data of the RNA-Seq analysis described above. The size of the detected bands was in the range of approximately 20–30 nt. Furthermore, larger RNA fragments with sizes of approximately 100 nt, 200 nt, 250 nt and above were visible (Fig 4B). Similar bands were also detected with a DNA probe, specific to sR-271, located on the AAV (-) strand (compare Fig 4C and 4B). Furthermore, a very faint band at a size around 25 nt was visible, corresponding to sR-271 (Fig 4C). In agreement with the varying read length distribution of sR-271 described above (see Fig 3C), it was not surprising that no sharp band could be detected in the gel for this small RNA. In contrast to sR-108 and sR-271, no AAV2-specific small RNA indicative of sR-1862 could be detected using a DNA/LNA probe (Fig 4D). The failure to validate sR-1862 by Northern blot analysis might be due to its 3- to 4-fold lower abundance compared to sR-108 and sR-271 (see Fig 3), probably lower than the detection limit of the used method. Unexpectedly, either probe (DNA and DNA/LNA) detected a strong Ad2-specific band of > 300 nt (Fig 4D). Even with highly stringent hybridization and washing conditions the bands persisted. An alignment to the Ad2 genome did not show a target sequence of the designed AAV2 probe. None of the AAV2-specific small RNAs was detected upon HSV co-infection, which was anticipated from their low abundance in RNA-Seq.

### Detection of human argonaute protein (Ago) co-immunoprecipitated RNAs

**Fig 5:**
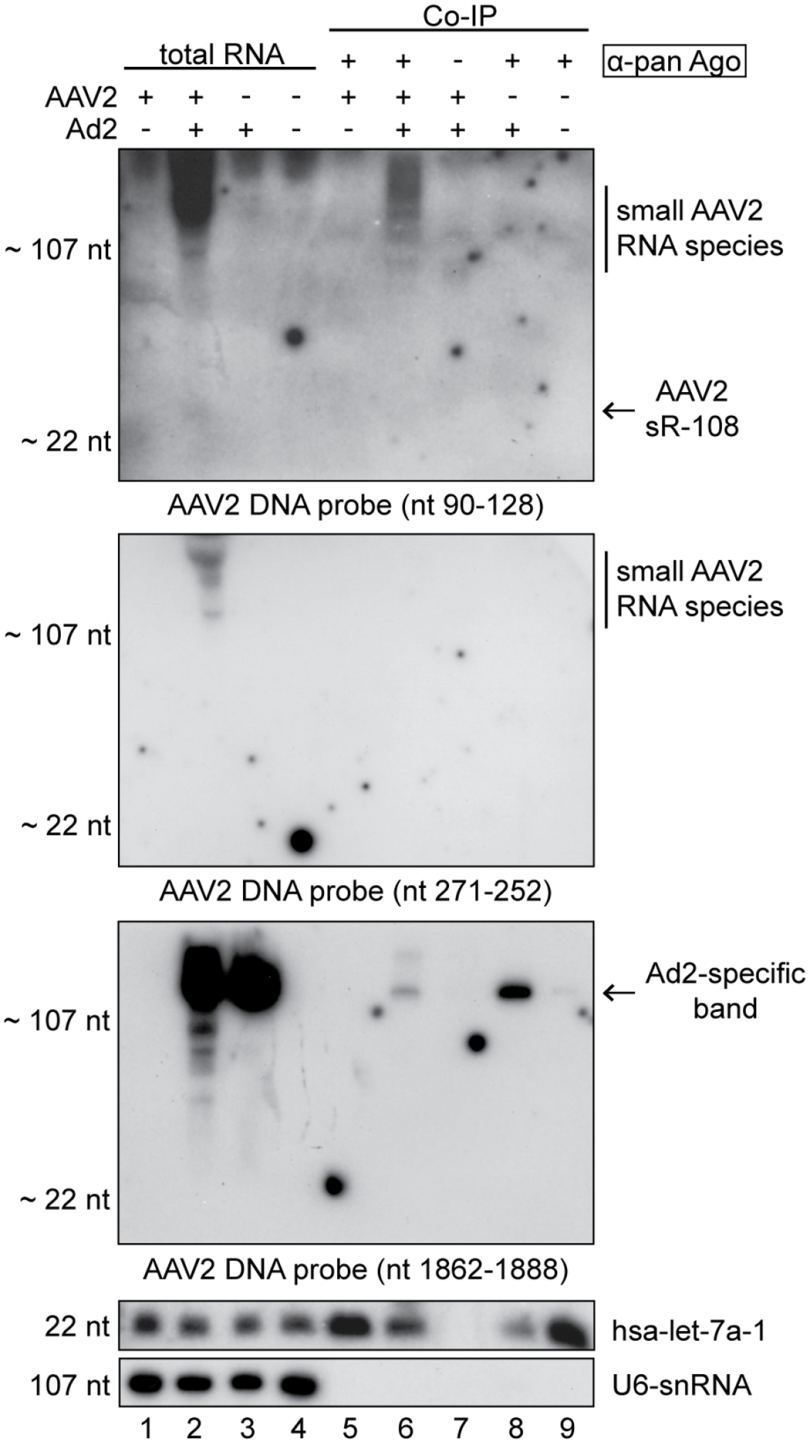
Human argonaute protein (Ago) co-immunoprecipitated RNAs of AAV2 infected cells. Northern blot analysis of DNase treated total RNA extracts or co-immunoprecipitated Ago-RNA complexes as indicated from HeLa cells, either mock infected, or infected with AAV2, AAV2 and Ad2, or Ad2, harvested 27 hpi. For co-immunoprecipitation, cell lysates were incubated over night with anti-pan Ago mAb or anti-Rep mAb 76–3 antibodies coupled to protein G agarose beads, respectively, as outlined in the methods. Northern blot analysis of purified RNAs was performed as described in Fig 4, with radiolabeled DNA probes directed against the AAV2-specific small RNAs sR-108, sR-271 or sR-1862, the human miRNA hsa-let-7a or the cellular U6-snRNA, respectively, as indicated. Note that instead of the human anti-pan Ago antibody an unrelated antibody (anti-Rep76-3) was used in lane 7 for co-immunoprecipitation.

To determine whether sR-108, sR-271 and sR-1862 represent functional miRNAs, their loading into the RNA-induced silencing complex (RISC) was studied. Ago2 is a component of RISC, which binds the mature miRNA and thus enables mRNA target recognition. We performed co-immunoprecipitations (Co-IP) of human Ago2 complexes with total RNAs from infected cells, using an antibody that detects the human argonaute proteins 1 to 4. Subsequently, the RNAs isolated from the precipitates were analyzed on Northern blots using probes directed against the AAV2-specific small RNAs sR-108, sR-271 or sR-1862, respectively. Probes directed against the human miRNA hsa-let-7a and the cellular U6-snRNA, a component of the splicosome, were used as controls. The human miRNA hsa-let-7a-1 could be detected in all total RNA and Co-IP RNA extracts, with the exception of co-immunoprecipitated RNAs in lane 7 for which an unrelated control antibody was used (Fig 5). In the AAV/Ad2 co-infected and Ad2 infected Co-IPs, hsa-let-7a-1 was reduced (Fig 5, lanes 6 and 8), in agreement with data that adenovirus down-regulate human hsa-let-7a [40] and blocks the RNAi pathway [22]. Since the U6-snRNA is not incorporated into RISC, it is only detectable in total RNA extracts (Fig 5, lanes 1 to 4). In contrast to hsa-let-7a, AAV-specific small RNAs could not be detected after immunoprecipitation of Ago2 complexes with any of the probes for sR-108, sR-271, or sR-1862 (Fig 5). Obviously, the identified small RNAs do not represent functional miRNAs. Instead, these RNAs may belong to another class of small non-coding regulatory RNAs, or are alternatively processed.

### Alignment of the small AAV2 RNAs to other AAV serotypes

To evaluate the relevance of the newly identified small RNAs the genomes of AAV serotypes 1 to 7 were aligned and compared to the AAV2 sequences of sR-108, sR-271 and sR-1862 (S1 Fig). The small AAV2 RNAs sR-108 and sR-1862 showed a high degree of conservation among the AAV serotypes, with the exception of AAV5, the most distantly related member. AAV2 sR-271 on the AAV (-) strand displayed some variations from other AAV serotypes in the central part of the small RNA.

### Effect of AAV2 on the expression levels of cellular miRNAs

**Fig 6:**
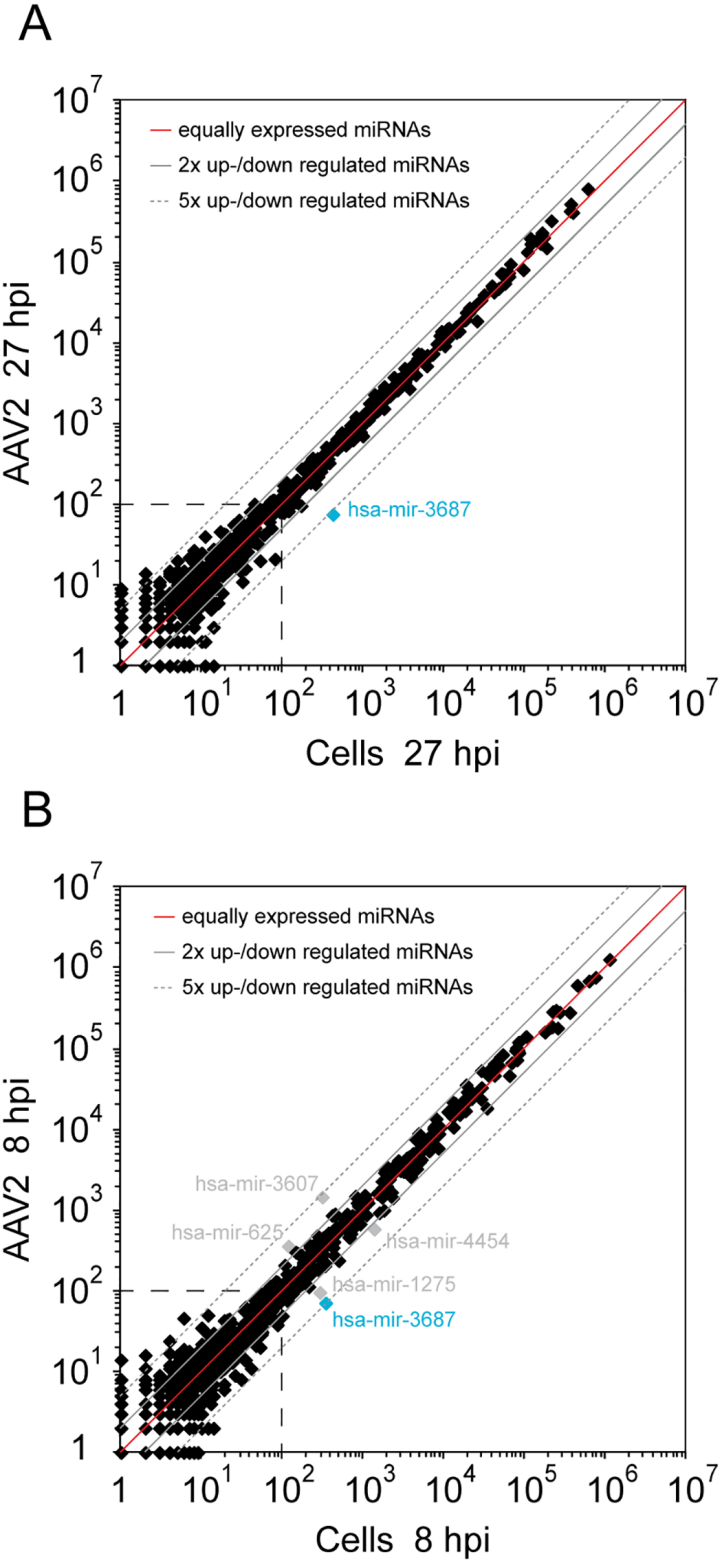
Effect of AAV2 infection on the expression of cellular miRNAs. (A) The expression levels of 979 described cellular miRNAs were analyzed by small RNA-Seq analysis in AAV2 infected versus uninfected HeLa cells at 27 hpi. Dots near the red line represent equally expressed miRNAs in both data sets, the solid gray lines mark 2-fold and the dashed gray lines 5-fold up- or down-regulation of miRNAs. The most highly up- or down-regulated miRNAs were highlighted and specified. Cellular miRNAs below a level of 100 read counts, within the dashed square, were not considered as representative. (B) Expression level of 979 described cellular miRNAs in AAV2 infected versus uninfected HeLa cells 8 hpi, displayed as in (A).

To assess whether AAV2 infection has an effect on the cellular miRNA expression profile, the read counts of 979 cellular miRNAs were determined, that are listed in the miRNA database (miRBase) [44]. Their expression levels determined by small RNA-Seq in AAV2 infected cells 8 hpi, 27 hpi were compared to those in uninfected cells (Fig 6). In addition, cellular miRNA regulation by lytic AAV replication in the presence of either helper virus was analyzed (S2 Fig). In the light of known extensive effects of either Ad or HSV on host cell transcription, miRNA effects cannot be readily attributed to lytic AAV infection. In the case of AAV infection alone the majority of cellular miRNAs were unaltered within a 2- to 5-fold range of regulation. One single human miRNA, hsa-mir-3687, was down-regulated slightly above 5-fold upon AAV infection (Fig 6A and 6B). To evaluate the significance of hsa-mir-3687 regulation the program "TargetScan" for miRNA target prediction was used and putative target genes were searched [45]. Eight human genes were predicted as potential targets of hsa-mir-3687, with MTA2 (metastasis associated 1 family, member 2) as the most likely candidate. However, the RNA expression levels of the predicted cellular target genes retrieved from our previously total RNA-Seq analysis performed under the same conditions were unaltered. Thus, AAV infection even at high MOIs does not significantly affect cellular miRNAs. This result extends earlier findings from microarray-based RNA screens demonstrating that AAV infection has no major effect on host mRNA expression [46]. The absence of miRNA induced transcriptional effects upon AAV infection further underlines the long-held notion that AAV infection is largely apathogenic.

## Discussion

In this report we present the first comprehensive analysis of AAV-encoded small RNAs from infected cells using Illumina small RNA-Seq. Whereas the cellular miRNA profile was largely unchanged by AAV infection, previously unrecognized small AAV RNAs were detected. Their abundance, AAV serotype conservation and predominant localization in close proximity to the established AAV promoters, p40 and p5, calls for roles as transcriptional regulators during latency and active replication.

### Putative generation of the small AAV2 RNAs

Based on their location we postulate that sR-108 and sR-271 are generated from the recently identified anti-p5 promoter, whereas sR-1862 is expressed from the p40 promoter. We recently detected the novel anti-p5 promoter on the AAV (-) strand [11]. The previously performed total RNA-Seq designed to detect long AAV transcripts led to its discovery.

**Fig 7:**
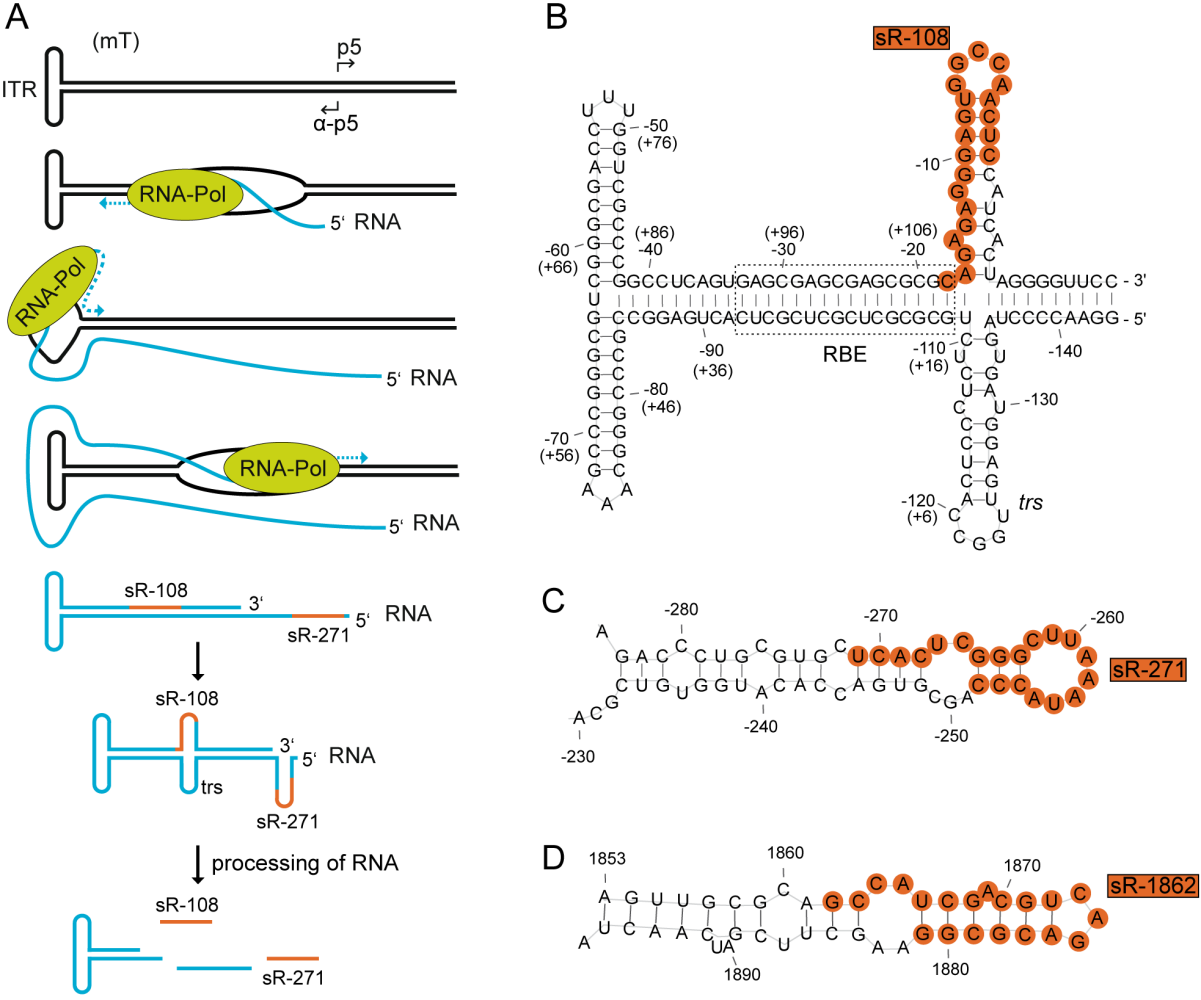
Model for the putative generation of the AAV2 small RNAs derived from the ITR and secondary structure formation. (A) A model for the putative generation of AAV2-specific small RNAs located within and close to the ITR is given. RNA transcription is initiated from the previously identified anti-p5 promoter on the AAV2 (-) strand. The RNA polymerase reads through the AAV2-ITR generating a transcript (highlighted in blue) that reforms the T-shape structure of the ITR. Furthermore, this transcript is able to form additional hairpin loops, which might be recognized by specific processing enzymes that generate the small RNAs sR-271 and sR-108 (highlighted in red). (B) Secondary structure formation of the AAV2-ITR RNA. Depicted are the nucleotide positions of the (-) and in brackets of the (+) strand. Indicated are the Rep binding element (RBE) framed in a dashed rectangle and the terminal resolution site (*trs*) at nucleotide position -125 (+1). The newly identified small RNA sR-108 ranging from nucleotide position 108 to 128 on the AAV (+) strand is highlighted in red. (C) Secondary structure formation of AAV2 RNA nucleotides 284 to 230 on the (-) strand. The newly identified small RNA sR-271 ranging from nucleotide position 271 to 252 on the AAV (-) strand is highlighted in red. (D) Secondary structure formation of AAV2 RNA nucleotides 1853 to 1897 on the (+) strand. The newly identified small RNA sR-1862 ranging from nucleotide position 1862 to 1881 on the AAV (+) strand is highlighted in red. Secondary structure prediction was generated using RNAfold.

In order to initiate transcription, second-strand synthesis of the AAV ssDNA genome is required [47]. Thereby a monomer turnaround structure (mT) is generated with the ITR connected to both complementary strands (Fig 7A). RNA polymerase can proceed through the ITR as shown recently for AAV mutants lacking the authentic polyA signal [48]. By continuing transcription on the opposite AAV strand double-stranded RNA can be formed (Fig 7A) as shown recently for the related parvovirus, minute virus of mice (MVM) [49]. The postulated AAV-ITR precursor transcript would comprise the two small RNAs, sR-108 and sR-271. Alternatively, RNA transcription could also be initiated independently at the ITR to yield the RNAs on the AAV (+) strand (sR-108). Transcripts mapping to the AAV2-ITRs have been reported previously [11, 50, 51], but neither 5’ ends nor function have been further investigated. For AAV5, the 5' end of the analogous transcripts could be mapped to nucleotide position 142 [52]. Small RNAs are often processed from longer precursor RNAs by various enzymes [53]. Due to the structure of the ITR the RNA product will automatically adopt a T-hairpin loop secondary structure (Fig 7A and 7B). An additional hairpin loop is generated at the terminal resolution site (*trs*) during Rep-dependent, strand-specific nicking of the AAV genome required to resolve the ssDNA genomes during AAV DNA replication [54, 55]. Recently, AAV2 Rep was shown to also bind to ITRs composed of RNA [48]. Nicking of the *trs* RNA-bound Rep, would result in the here-described small RNAs starting at nucleotide position 1 or 125, respectively (Fig 3, sR-1 and sR-125). Furthermore, genome-wide AAV integration analysis implied that Rep-induced nicking within the human genome does not necessarily require a *trs* adjacent to the Rep binding element (RBE) [2], leaving the possibility of Rep endonucleolytic attack at other sites. Similar to the ITR, the region near the anti-p5 promoter potentially allows formation of a RNA secondary structure (Fig 7C). The sequence of sR-271 partially overlaps with the p5 RBE and with the p5 TATA-box on the complementary strand. Since binding of Rep was also shown on the RBE within the p5 promoter [56], Rep could also play a role in RNA processing at this site.

Transcription of the small RNA sR-1862, located a few nucleotides downstream of the p40 TSS (nt 1853) could also lead to a secondary structure (Fig 7D). Recently, we have identified this region as *cis*-regulatory element for the inhibition of recombinant adenovirus replication [12]. Here we show by small RNA-Seq that p40-initiated transcripts although highly heterogeneous (Fig 3A), mostly represent sR-1862.

Interestingly, in all stem loop models the small RNAs, sR-108, sR-271 and sR-1862, span sequences, which cover both sides of the stem loop including the terminal loop (Fig 7B–7D). It is apparent that a processing mechanism other than the canonical miRNA processing pathway is required to generate these small RNAs, since Dicer regularly removes the terminal loop for miRNA generation.

### Potential role of small AAV2 RNAs

Typically, virus encoded small non-coding RNAs are expressed either to support virus propagation by generating beneficial conditions or to evade the cellular immune response [21]. The majority of identified small RNAs represent microRNAs, but also other types of small RNAs have been described [57–59]. For the parvovirus family including AAV, small RNAs have not yet been reported. The here identified AAV-specific small RNAs are candidate regulators to adapt to the environment of the host cell and/or co-replicating helper viruses.

The functional role of the small RNAs located within the ITRs are difficult to analyze by genetic means, due to an overlap with elements required for DNA replication and/or virus packaging [60, 61]. A series of previously described mutants in these elements showed a replication- and/or packaging-deficient phenotype [62, 63]. In addition to affecting the functions of the AAV RBE and *trs*, the described mutations might have inadvertently abolished the generation of the newly detected small RNAs.

In a recent study we have shown that short transcripts initiated at the anti-p5 promoter are generated [11]. Due to the heterogeneous character of these anti-p5 transcripts, we concluded a mechanism reminiscent of divergent transcription from the AAV p5 promoter. In agreement with that, small RNA-Seq showed several hotspots of small AAV2 RNAs initiated from the anti-p5 promoter with various 5’ ends (Fig 3). The short antisense transcripts generated during divergent transcription are believed to play a role in gene regulation by modulating promoter activity [64]. Similarly, the here identified small RNAs sR-271, sR-262 and sR-241 might play a role in AAV p5 promoter activity regulation.

In addition, a heterogeneous group of small AAV p40 promoter-associated transcripts has been described [12]. A mechanism of RNA polymerase II pausing accompanied with the inhibition of recombinant adenoviral replication was postulated. The here identified small RNA sR-1862 starts next to the p40 TSS (nt 1853) and might be a likely candidate for adenoviral inhibition. On the other hand, sR-1862 could also represent a transcription initiation RNA (tiRNA). These small RNAs of 18 nt in size are typically located just downstream the TSS of promoters and are generated by RNA polymerase II backtracking and cleavage by TFIIS. For eukaryotes, tiRNAs have been described to be associated with highly expressed transcripts [65]. Driving the expression of AAV capsid proteins the AAV p40 promoter is extremely active late during AAV replication.

The complex regulation of the AAV life cycle has not yet been fully unraveled, particularly not *in vivo*. The discovery of the small AAV RNA species some derived from transcripts from within the AAV-ITRs and associated with the major AAV promoters adds a new piece of information to the unsolved puzzle of the AAV switch from latency to productive replication. In the absence of a suitable animal model to study the AAV biology *in vivo*, systems biology approaches to study viral interaction with the host cell transcriptome offer detailed insight onto potentially pathogenic host cell effects. Surprisingly and in contrast to most other virus infections studied, AAV infection neither induces own miRNAs nor regulates host cell miRNA levels to any significant extend. These findings are in line with earlier microarray-based cellular mRNA analysis, where AAV infection showed virtually no regulation [46]. Together the data underline the notion of AAV’s apathogenicity. Further studies particularly *in vivo* will be required to unravel the role of the newly identified, obviously regulatory small AAV RNAs for the AAV life cycle.

## Supporting Information

S1 Fig Alignment of AAV2-specific small RNAs for AAV serotypes 1 to 7. The small AAV2-specific RNAs sR-108, sR-271 and sR-1862 were aligned to the corresponding sites of AAV serotypes 1 to 7. Gray areas represent conserved nucleotide regions; white areas indicate aberrations to the AAV2 small RNAs in the given AAV serotype.
(TIF)

S2 Fig Effect of lytic AAV2 infection on expression levels of cellular miRNAs. (A) The expression levels of 979 described cellular miRNAs were analyzed by small RNA-Seq analysis in AAV2/Ad2 co-infected versus uninfected HeLa cells at 27 hpi. Dots near the red line represent equally expressed miRNAs in both data sets; the solid gray lines mark 2-fold and the dashed gray lines 5-fold up- or down-regulation of miRNAs. The most highly up- or down-regulated miRNAs were highlighted and specified. Cellular miRNAs below a level of 100 read counts, within the dashed square, were considered as not representative. (B-D) Expression level of 979 described cellular miRNAs in (B) AAV2/HSV1 co-infected versus uninfected HeLa cells 8 hpi; (C) Ad2 infected versus uninfected HeLa cells 27 hpi; (D) HSV1 infected versus uninfected HeLa cells 8 hpi, displayed as in (A).
(TIF)

S1 Table Oligonucleotide sequences used for Northern blot analysis. (DOC)

S2 Table Small RNA-Seq analysis—Assignment of the reads to the species from which they originate (gradually allowing zero, one and two mismatches per read). (DOCX)

S3 Table Small AAV2 RNA list. Listed are the top hundred AAV2-specific small RNA candidates detected by small RNA-Seq analysis in the presence of the helper viruses Ad2 and HSV1, respectively. Given are genomic location, read counts, median read length and sequence of the respective small AAV RNAs.
(XLS)
