# Supplementary figures and images for: Comprehensive Small RNA-Seq of Adeno-Associated Virus (AAV)-Infected Human Cells Detects Patterns of Novel, Non-Coding AAV RNAs in the Absence of Cellular miRNA Regulation
Source: PLoS One. 2016 Sep 9;11(9):e0161454. doi: 10.1371/journal.pone.0161454 (PMC5017669; doi:10.1371/journal.pone.0161454)

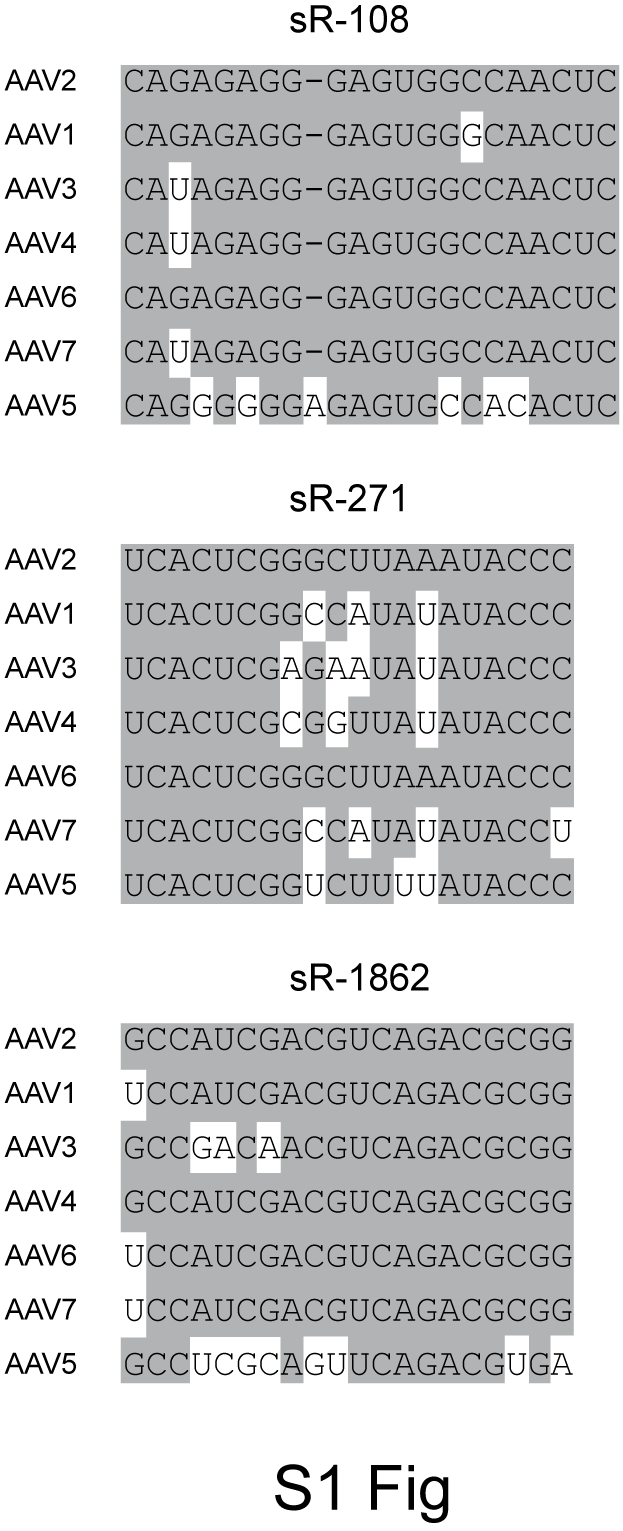

Supplement: S1 Fig — The small AAV2-specific RNAs sR-108, sR-271 and sR-1862 were aligned to the corresponding sites of AAV serotypes 1 to 7. Gray areas represent conserved nucleotide regions; white areas indicate aberrations to the AAV2 small RNAs in the given AAV serotype. (TIF) [file pone.0161454.s001.tif]

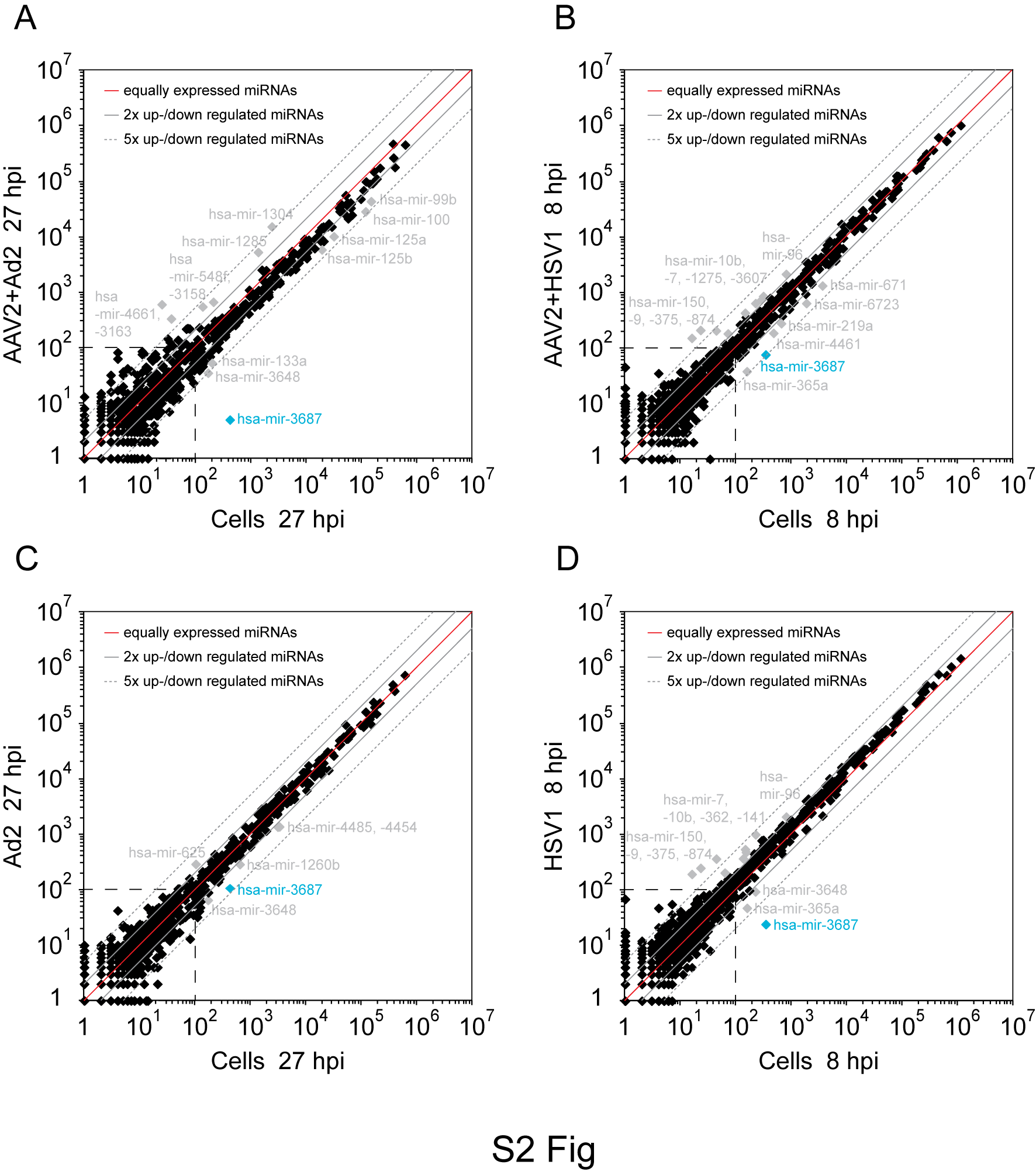

Supplement: S2 Fig — (A) The expression levels of 979 described cellular miRNAs were analyzed by small RNA-Seq analysis in AAV2/Ad2 co-infected versus uninfected HeLa cells at 27 hpi. Dots near the red line represent equally expressed miRNAs in both data sets; the solid gray lines mark 2-fold and the dashed gray lines 5-fold up- or down-regulation of miRNAs. The most highly up- or down-regulated miRNAs were highlighted and specified. Cellular miRNAs below a level of 100 read counts, within the dashed square, were considered as not representative. (B-D) Expression level of 979 described cellular miRNAs in (B) AAV2/HSV1 co-infected versus uninfected HeLa cells 8 hpi; (C) Ad2 infected versus uninfected HeLa cells 27 hpi; (D) HSV1 infected versus uninfected HeLa cells 8 hpi, displayed as in (A). (TIF) [file pone.0161454.s002.tif]
