# Supplementary material for: Comprehensive Small RNA-Seq of Adeno-Associated Virus (AAV)-Infected Human Cells Detects Patterns of Novel, Non-Coding AAV RNAs in the Absence of Cellular miRNA Regulation
Source: PLoS One. 2016 Sep 9;11(9):e0161454. doi: 10.1371/journal.pone.0161454 (PMC5017669; doi:10.1371/journal.pone.0161454)
Supplement: S1 Table — (DOC) [file pone.0161454.s003.doc]

**S1** Table. Oligonucleotide sequences used for Northern blot analysis.

| **sRNA name** | **Probe sequence 5’ → 3’ (source)** |
| --- | --- |
| sR-108 | 5’ GAGTTGGCCACTCCCTCTCTGCGCGCTCGCTCGCTCACT 3‘ (DNA) |
| sR-108 | 5’ GAGTTGGCC+ACTCCC+T+CTCT+GCGCG+C+TCGC+TCGC+TCACT 3’ (DNA/LNA) |
| sR-1862 | 5’ GAAGCTTCCGCGTCTGACGTCGATGGC 3‘ (DNA) |
| sR-1862 | 5‘ GAAGCTTC+C+GCGTC+TGACG+T+CGA+TGGC 3‘ (DNA/LNA) |
| sR-271 | 5’ GGGTATTTAAGCCCGAGTGA 3’ (DNA) |
| hsa-let-7a-1 | 5’ AACTATACAACCTACTACCTCA 3’ (DNA) |
| U6-snRNA | 5’ CACGAATTTGCGTGTCATCCTT 3’ (DNA) |

Underlined is the exact complementary sequence of the corresponding sRNA identified in the small RNA-Seq analysis.
