# Supplementary material for: Comprehensive Small RNA-Seq of Adeno-Associated Virus (AAV)-Infected Human Cells Detects Patterns of Novel, Non-Coding AAV RNAs in the Absence of Cellular miRNA Regulation
Source: PLoS One. 2016 Sep 9;11(9):e0161454. doi: 10.1371/journal.pone.0161454 (PMC5017669; doi:10.1371/journal.pone.0161454)
Supplement: S2 Table — (DOCX) [file pone.0161454.s004.docx]

**S2 Table. Small RNA-Seq analysis - Assignment of the reads to the species from which they originate (gradually allowing zero, one and two mismatches per read).**

| **Data**  **set** | **Total no. of reads** | **No. (%) of reads**  **≥ 16 nt** | **No. (%) of human reads^a^** | **No. (%) of small reads assigned to:** | | | **No. (%) of unknown reads^b^** |
| --- | --- | --- | --- | --- | --- | --- | --- |
|  |  |  |  | **AAV2** | **Ad2** | **HSV1** |  |
| **Cells**  **(27 hpi)** | 19,395,459 | 17,429,392  (89.9) | 16,672,836  (95.7) | 22  (<0.1) | 621  (<0.1) | 10  (<0.1) | 755,903  (4.3) |
| **AAV2**  **(27 hpi)** | 18,085,432 | 16,228,519  (89.7) | 15,441,982  (95.2) | 1,400  (<0.1) | 1,703  (<0.1) | 14  (<0.1) | 783,420  (4.8) |
| **AAV2**  **+ Ad2**  **(27 hpi)** | 21,333,021 | 18,814,505  (88.2) | 13,008,523  (69.1) | 328,917  (1.7) | 4,663,151  (24.8) | 20  (<0.1) | 813,894  (4.3) |
| **Ad2**  **(27 hpi)** | 24,841,074 | 24,202,341  (97.4) | 14,184,796  (58.6) | 29  (<0.1) | 9,214,482  (38.1) | 23  (<0.1) | 803,011  (3.3) |

| **Cells**  **(8 hpi)** | 21,851,591 | 20,985,998  (96.0) | 19,964,098  (95.1) | 24  (<0.1) | 544  (<0.1) | 18  (<0.1) | 1,021,314  (4.9) |
| --- | --- | --- | --- | --- | --- | --- | --- |
| **AAV2**  **(8 hpi)** | 20,375,107 | 20,022,269  (98.3) | 18,992,333  (94.9) | 176  (<0.1) | 791  (<0.1) | 16  (<0.1) | 1,028,953  (5.1) |
| **AAV2**  **+ HSV1**  **(8 hpi)** | 23,031,470 | 20,904,023  (90.8) | 19,108,356  (91.4) | 158,561  (0.8) | 7,867  (<0.1) | 127,303  (0.6) | 1,501,936  (7.2) |
| **HSV1**  **(8 hpi)** | 22,391,766 | 20,895,686  (93.3) | 19,600,347  (93.8) | 15  (<0.1) | 616  (<0.1) | 145,417  (0.7) | 1,149,291  (5.5) |

^a^Included are reads mapped to the human genome, transcriptome and miRNAs.

^b^Included are reads ≥ 16 nt that were unmappable during sequence mapping allowing two mismatches.
